# Use of Quantitative Electroencephalography to Inform Age- and Sex-Related Differences in NMDA Receptor Function Following MK-801 Administration

**DOI:** 10.3390/ph17020237

**Published:** 2024-02-11

**Authors:** Kimberly M. Holter, Alex D. Lekander, Bethany E. Pierce, L. Paul Sands, Robert W. Gould

**Affiliations:** Department of Translational Neuroscience, Wake Forest University School of Medicine, Winston-Salem, NC 27157, USA; kholter@wakehealth.edu (K.M.H.);

**Keywords:** electroencephalography (EEG), sex differences, schizophrenia, MK-801, gamma band power, aging

## Abstract

Sex- and age-related differences in symptom prevalence and severity have been widely reported in patients with schizophrenia, yet the underlying mechanisms contributing to these differences are not well understood. *N*-methyl-D-aspartate (NMDA) receptor hypofunction contributes to schizophrenia pathology, and preclinical models often use NMDA receptor antagonists, including MK-801, to model all symptom clusters. Quantitative electroencephalography (qEEG) represents a translational approach to measure neuronal activity, identify targetable biomarkers in neuropsychiatric disorders and evaluate possible treatments. Abnormalities in gamma power have been reported in patients with schizophrenia and correspond to psychosis and cognitive impairment. Further, as gamma power reflects cortical glutamate and GABA signaling, it is highly sensitive to changes in NMDA receptor function, and NMDA receptor antagonists aberrantly increase gamma power in rodents and humans. To evaluate the role of sex and age on NMDA receptor function, MK-801 (0.03–0.3 mg/kg, SC) was administered to 3- and 9-month-old male and female Sprague–Dawley rats that were implanted with wireless EEG transmitters to measure cortical brain function. MK-801-induced elevations in gamma power were observed in 3-month-old male and female and 9-month-old male rats. In contrast, 9-month-old female rats demonstrated blunted maximal elevations across a wide dose range. Importantly, MK-801-induced hyperlocomotor effects, a common behavioral screen used to examine antipsychotic-like activity, were similar across all groups. Overall, sex-by-age-related differences in gamma power support using qEEG as a translational tool to evaluate pathological progression and predict treatment response across a heterogeneous population.

## 1. Introduction

Decades of research have identified clear sex differences in schizophrenia relating to age of onset and symptom prevalence, severity, and overall treatment outcome [1,2]. For example, males have an earlier average age of onset, with most first-episode diagnoses occurring between 15 and 24, compared to between 20 and 29 years of age in females [1,3]. Age-related differences further extend to later in life as females represent over 75% of first-episode diagnosis between the ages of 45 and 50 [1,4], an age range that corresponds to the menopause transition [5]. Additionally, as it pertains to symptom prevalence and severity, males generally have an overall more severe course of illness with increased prevalence of negative symptoms and cognitive impairment; alternatively, female patients more commonly experience impulsivity and depressive symptoms [1,3]. However, few preclinical studies have investigated the underlying mechanisms contributing to this variability, hindering advancements in potential sex- and/or age-specific therapeutic approaches.

*N*-methyl-D-aspartate (NMDA) receptor hypofunction is a major contributing factor to the underlying pathology of schizophrenia [6,7,8,9]. NMDA receptor antagonists administered to healthy humans induced psychotic-like symptoms, including delusions and hallucinations as well as impaired learning and memory [10,11,12,13]. Further, NMDA receptor antagonists administered to patients with schizophrenia worsened psychotic symptoms [13,14]. Region-specific alterations in the gene and protein expression of NMDA receptor subunits have also been identified in post-mortem studies from patients with schizophrenia [15,16,17,18]. Thus, several genetic and pharmacological animal models have been developed to parallel the NMDA receptor hypofunction identified in the human patient population. NMDA receptor antagonists including MK-801 are frequently administered to rodents to model all symptom clusters, including positive, negative, and cognitive symptoms [8].

Given the heterogeneity of symptom profile and severity associated with schizophrenia, recent drug development efforts have demonstrated a high failure rate, emphasizing a need for more effective biomarker strategies that translate from basic mechanistic research to implementation in clinical trials [19,20,21]. Electroencephalography (EEG) can be used as a biomarker for normal and aberrant brain function and can be widely applied across species. Abnormalities in neuronal oscillatory activity detected using quantitative EEG (qEEG), including impairments in the resting state and evoked cortical activity in the high-frequency gamma power range, have been widely reported in patients with schizophrenia and have strongly correlated with positive symptoms and cognitive impairment [22,23,24,25]. Furthermore, NMDA receptors are located on both glutamatergic pyramidal cells and GABAergic parvalbumin (PV)-containing interneurons. As gamma power reflects the balance of cortical glutamate and GABA activity, both genetic and pharmacological studies suggest that it is highly sensitive to NMDA receptor manipulation on PV interneurons [26,27,28]. NMDA receptor antagonists including MK-801, ketamine, and PCP also affect gamma power in both humans and rodents, supporting the relevance of this waveform as a translational biomarker in schizophrenia [27,29,30,31]. Moreover, qEEG can be used in preclinical and human research studies to measure cortical activity and is sensitive to factors including age [32] and sex differences [33] that may contribute to variability in the overall course of illness. Ultimately, qEEG serves as a valuable tool to inform pathophysiological changes with strong utility in pharmacotherapeutic development [19].

Herein, we sought to examine the impact of both sex and age on NMDA receptor function using EEG in freely moving 3-month-old and 9-month-old male and female rats at baseline and following MK-801 administration. We analyzed MK-801-induced changes across a full power spectrum from low- to high-frequency activity, with a focus on high gamma power (50–100 Hz). We also assessed changes in locomotor activity, as NMDA receptor antagonist-induced hyperlocomotion is commonly used in preclinical studies as a behavioral readout to predict antipsychotic-like efficacy for positive symptoms. In line with differences reported in the aforementioned clinical studies, we hypothesized that both sex- and age-related factors would influence MK-801-induced changes in gamma power. Consistent with prior studies, the present results show that MK-801 produced changes in multiple frequency bands in 3-month-old male rats with significant elevations in high gamma power [50–100 Hz]. However, complex differences in both the magnitude and overall temporal pattern of the effects were identified across all groups. Notably, the 9-month-old female rats displayed a vastly different profile than the other tested groups, demonstrating both an elevated baseline high gamma power and blunted peak effects on high gamma power following MK-801 administration. Further, as MK-801 commonly displays a biphasic effect on gamma power, the higher tested doses of MK-801 produced divergent effects across groups. Overall, given the strong group differences, these studies support qEEG as a biomarker sensitive to individual variability and suggest a sex-by-age interaction influencing NMDA receptor function.

## 2. Results

### 2.1. Nine-Month-Old Female Rats Had Higher Baseline Relative High Gamma Power

**Figure 1:**
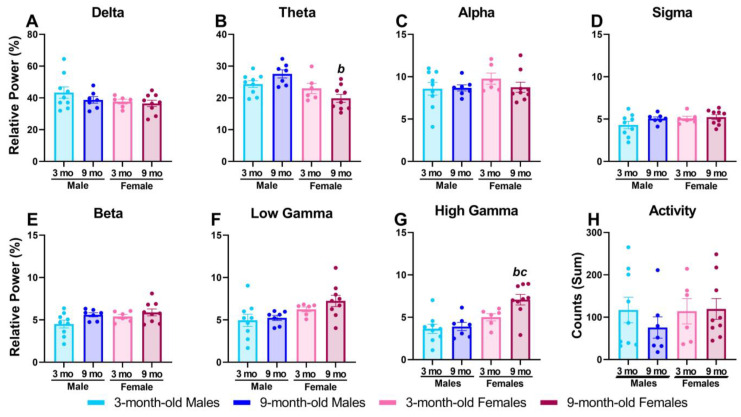
Nine-month-old female rats had higher baseline relative high gamma power. qEEG data are shown as a group mean ± SEM of relative power during the 90 min baseline period on each individual’s respective vehicle day. Locomotor activity is expressed as summed activity counts during the baseline period. Data are averaged across the delta [0.5–4 Hz] (**A**), theta [4–8 Hz] (**B**), alpha [8–12 Hz] (**C**), sigma [12–16 Hz] (**D**), beta [16–24 Hz] (**E**), low gamma [30–50 Hz] (**F**), and high gamma [50–100 Hz] (**G**) frequency bands and summed activity (**H**) to compare 3-month-old male (*n* = 9) and female (*n* = 6) and 9-month-old male (*n* = 7) and female (*n* = 9) rats; *p* < 0.05; b, compared to 9-month-old male rats; c, compared to 3-month-old female rats; circles represent individual datapoints.

Prior to assessing the effects of MK-801, we compared group averages in relative power for all frequency bands (Figure 1A–G). There was a main effect of sex (F_1, 27_ = 12.16, *p* < 0.005) and a significant sex-by-age interaction (F_1, 27_ = 5.915, *p* < 0.05) in baseline theta power; post hoc analyses revealed a significant difference between 9-month-old male and 9-month-old female rats, where 9-month-old females had significantly lower baseline theta power (Figure 1B). Additionally, there was a main effect of sex (F_1, 27_ = 7.222, *p* < 0.05) in baseline low gamma power, but post hoc analysis showed no significant group differences (Figure 1F). Lastly, as shown in Figure 1G, there was a main effect of both sex (F_1, 27_ = 16.09, *p* < 0.001) and age (F_1, 27_ = 4.319, *p* < 0.05) on baseline high gamma power, and post hoc analysis revealed 9-month-old female rats had a significantly higher baseline than 3-month-old female and 9-month-old male rats. There were no main effects or significant interactions for delta, alpha, sigma, or beta. Additionally, baseline absolute power was analyzed, and no significant differences between groups were found at any frequency band or in total power (Figure S1). Lastly, summed locomotor activity during the 90 min baseline period was not different between groups (Figure 1H).

### 2.2. MK-801 Differentially Affected Spectral Frequency Distribution in 9-Month-Old Female Rats

**Figure 2:**
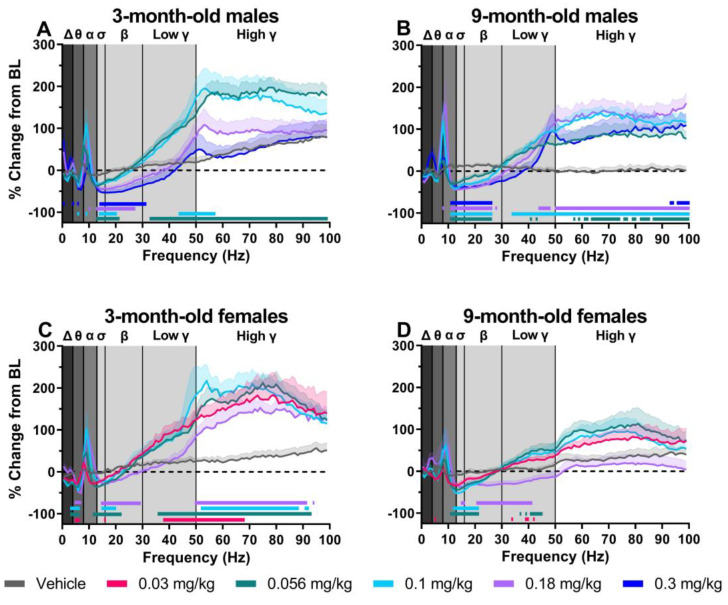
MK-801 differentially affected spectral frequencies in 9-month-old female rats. Data are shown as a group mean ± SEM presented in 1 Hz bins expressed as the average percent change from baseline (average of each individual’s 90 min baseline just prior to compound administration) during the 30–90 min post-dosing period. Gray vertical bars represent frequency bands (delta, Δ 0.5–4 Hz; theta, θ 4–8 Hz; alpha, α 8–13 Hz; sigma, σ 13–15 Hz; beta, β 13–30 Hz; low gamma, γ 30–50 Hz; high gamma, γ 50–100 Hz). All tested doses were examined within each individual group: 3-month old male rats (*n* = 8–9) (**A**), 9-month-old male rats (*n* = 7–8) (**B**), 3-month-old female rats (*n* = 6) (**C**), and 9-month-old female rats (*n* = 9) (**D**). Horizontal colored lines matching the color of respective doses represent frequencies at which MK-801-treated groups were significantly different from vehicle-treated groups (*p* < 0.05) (**A**–**D**).

The NMDA receptor antagonist MK-801 produced dose-dependent changes on spectral power when examined as an average of the 30–90 min post-dosing period. All data are expressed as the percent change from the 90 min baseline directly prior to MK-801 administration. In all groups, there was a main effect of MK-801 dose and frequency and a significant dose-by-frequency interaction (Figure 2A–D; for statistics, see Table 1). In 3-month-old male rats, post hoc analysis revealed significant dose-dependent changes in relative power when compared to the vehicle treatment. Significant decreases were reported in frequencies corresponding to the delta (0.3 mg/kg), theta (0.1 and 0.3 mg/kg), sigma (all tested doses), and beta (all tested doses) frequency bands and significant increases were reported in frequencies associated with the alpha (0.1 and 0.18 mg/kg) and low- and high gamma (0.056 and 0.1 mg/kg) frequency bands (Figure 2A; for statistics, see Table 1). Similar changes were seen in 9-month-old male rats, with post hoc analysis revealing significant, dose-dependent decreases in frequencies corresponding to the sigma and beta frequency bands (all tested doses) and increases in frequencies corresponding to the alpha (all tested doses), low gamma (0.056–0.18 mg/kg), and high gamma (all tested doses) frequency bands (Figure 2B; for statistics, see Table 1). As shown in Figure 2C, 3-month old females also had dose-dependent decreases in frequencies corresponding to delta (0.056 and 0.1 mg/kg), theta (all tested doses), alpha (0.056–0.18 mg/kg), sigma (all tested doses), and beta (0.056–0.18 mg/kg) and increases in frequencies corresponding to low gamma (0.03 and 0.056 mg/kg) and high gamma (all tested doses; for statistics, see Table 1). Lastly, 9-month-old females had similar decreases in frequencies corresponding to the theta (0.03 mg/kg), sigma (0.056–0.18 mg/kg), and beta (0.056–0.18 mg/kg) frequency bands and similar increases in frequencies corresponding to the alpha frequency band (0.056 and 0.1 mg/kg; Figure 2D). By contrast, a unique profile compared to the other three groups was identified among frequencies corresponding to the low- and high gamma frequency bands. While significant increases in low gamma frequencies were found following administration of 0.03 and 0.056 mg/kg, there was a significant decrease following administration of 0.18 mg/kg. Furthermore, there were no significant changes in the frequencies associated with the high gamma frequency band (Figure 2D; for statistics, see Table 1).

### 2.3. Nine-Month-Old Females Display Lower MK-801-Induced Elevations in High Gamma Power Compared to Other Groups

**Figure 3:**
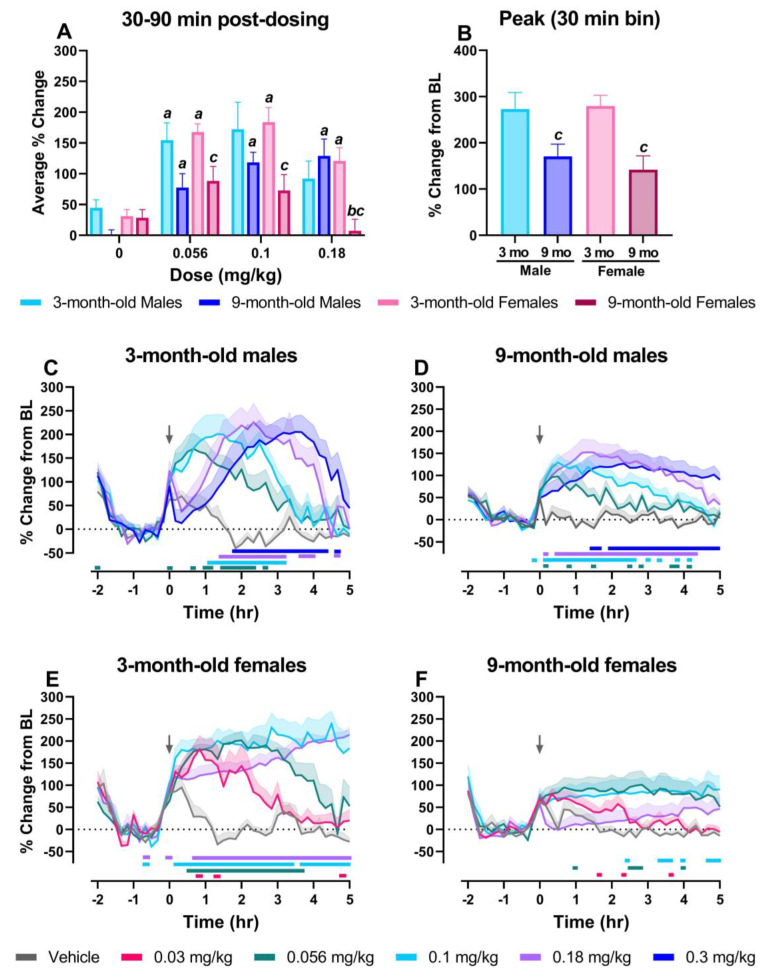
Nine-month-old females displayed lower MK-801-induced elevations in high gamma power compared to other groups. For direct group comparisons, each individual’s percent change from baseline in the 30–90 min post-dosing period (**A**) or maximum percent change from baseline over the full 5 h post-dosing period (**B**) was averaged and graphed as a group mean ± SEM. The effects of MK-801 on high gamma power over time are displayed as group means ± SEM of the percent change from baseline in 10 min bins across the 7 h recording period for 3-month-old male (*n* = 8–9) (**C**), 9-month-old male (*n* = 7–8) (**D**), 3-month-old female (*n* = 6) (**E**), and 9-month-old female rats (*n* = 9) (**F**). MK-801 was administered at time point 0, denoted by an arrow. On the x-axis, -2 corresponds to ZT 0, and 5 corresponds to ZT 7 (**C**–**F**). In (**A**,**B**), *p* < 0.05; *a*, compared to the group’s respective vehicle condition; *b*, compared to 9-month-old male rats; *c*, compared to 3-month-old female rats. In (**C**–**F**), horizontal colored lines matching the respective dose color represent the 10 min bins at which MK-801-treated groups were significantly different from vehicle-treated groups (*p* < 0.05).

Given the strong relationship between schizophrenia, NMDA receptor antagonists and changes in resting-state gamma power, MK-801-induced changes on the high gamma power band between groups was the primary focus of these studies. For direct group comparisons, we first assessed the average % change during the 30–90 min post-dosing period for the vehicle and the 0.056–0.18 mg/kg MK-801 dose range. A three-way mixed ANOVA revealed a main effect of dose (F_2.43, 66.54_ = 22.59; *p* < 0.0001) and age (F_1, 28_ = 9.830; *p* < 0.005) and a significant dose-by-sex-by-age interaction (F_3, 82_ = 4.593; *p* < 0.05) (Figure 3A). Two-way ANOVAs were then conducted to assess effects of dose and age in males and females. In males, there was a main effect of dose (F_2.662, 38.16_ = 11.25, *p* < 0.0001), whereas in females there was a main effect of dose (F_1.671, 21.72_ = 21.42, *p* < 0.0001) and age (F_1.671, 21.72_ = 21.42, *p* < 0.05) and a significant dose-by-age interaction (F_3, 39_ = 5.987= *p* < 0.05). The 3-month-old male rats showed significant elevations from their respective vehicle condition at the 0.056 mg/kg dose and 9-month-old male rats experienced significant elevations at the 0.056, 0.1, and 0.18 mg/kg doses. Interestingly, while 3-month-old female rats showed significant elevations at all doses (0.056–0.18 mg/kg) relative to their respective vehicle condition, 9-month-old female rats did not show significant elevations compared to vehicle and displayed a significantly lower percent change from baseline compared to 3-month-old female rats at all doses (0.056–0.18 mg/kg) (Figure 3A). Next, two-way ANOVAs were conducted to assess effects of dose and sex at each age (3- and 9-month-old). A main effect of dose was identified in the 3-month-old rats (F_2.416, 31.40_ = 14.85, *p* < 0.0001) whereas in 9-month-old rats there was a main effect of dose (F_1.941, 27.83_ = 10.38, *p* < 0.001) and a significant dose-by-sex interaction (F_3, 43_ = 9.472, *p* < 0.0001). Post hoc analyses revealed that 9-month-old female rats had a significantly lower percent change from baseline than 9-month-old male rats at the 0.18 mg/kg dose of MK-801 (Figure 3A). Next, the peak % change from baseline in high gamma power over the 5 h post-dosing period was compared for each group (Figure 3B). A two-way ANOVA revealed a main effect of age (F_1, 28_ = 14.63, *p* < 0.001); both 9-month-old males (170.65 ± 75.07%) and 9-month-old females (141.75 ± 90.42%) experienced a lower peak % change compared to 3-month-old males (272.82 ± 107.66%) or 3-month-old females (279.39 ± 57.59%), respectively.

We then examined the dose-dependent effects of MK-801 on high gamma power over time in 10-min bins during the 7 h recording period in each group. In all groups, there was a main effect of MK-801 dose and time and a significant dose-by-time interaction (Figure 3C–F; for statistics, see Table 2). In 3-month-old male rats, post hoc analysis revealed significant increases in high gamma power across all tested doses of MK-801 (0.056–0.3 mg/kg) when compared to vehicle (Figure 3C). While the largest increases in high gamma power following administration of 0.056 mg/kg and 0.1 mg/kg MK-801 occurred at ~1–2 h post-administration and dissipated at around 3 h, the largest increases at the higher doses, 0.18 and 0.3 mg/kg, were delayed; these were observed between 2 and 4 h post-administration and had dissipated by hour 5 (Figure 3C; for statistics, see Table 2). Similarly, post hoc analysis in 9-month-old males revealed significant increases in high gamma power relative to the vehicle treatment following administration of all tested doses. However, unlike 3-month-old males, the largest elevations for all tested doses occurred between 30 min and 2 h following MK-801 administration and either dissipated by hour 5 (0.056–0.18 mg/kg) or remained stable for the full 5 h period (0.3 mg/kg) (Figure 3D, for statistics see Table 2).

Post hoc analysis showed 3-month-old female rats also experienced significant increases in high gamma power relative to vehicle at all tested doses (Figure 3E). The greatest increases for 0.03 mg/kg, 0.056 mg/kg, and 0.1 mg/kg occurred within the first 2 h following MK-801 administration, and, while elevations for 0.03 and 0.056 mg/kg subsided by the end of the 5 h post-dosing period, elevations following 0.1 mg/kg persisted for the full recording period. Administration of 0.18 mg/kg was followed by delayed elevations, and gradual increases were still observed at the end of the 5 h recording period (Figure 3E, for statistics, see Table 2). As shown in Figure 3F, a unique pattern was identified in 9-month-old female rats; few significant increases relative to vehicle were identified, and elevations for all doses were blunted compared to other groups across the full 5 h post-dosing period. The highest dose administered, 0.18 mg/kg, produced no significant elevations across the full recording period (Figure 3F, for statistics, see Table 2).

Time-course graphs and direct comparisons between groups in the 30–90 min post-administration period for spectral frequency bands below 50 Hz are shown in Supplementary Figures S2–S6 and Supplementary Tables S2 and S3.

### 2.4. MK-801 Dose-Dependently Increased Locomotor Activity in All Groups

**Figure 4:**
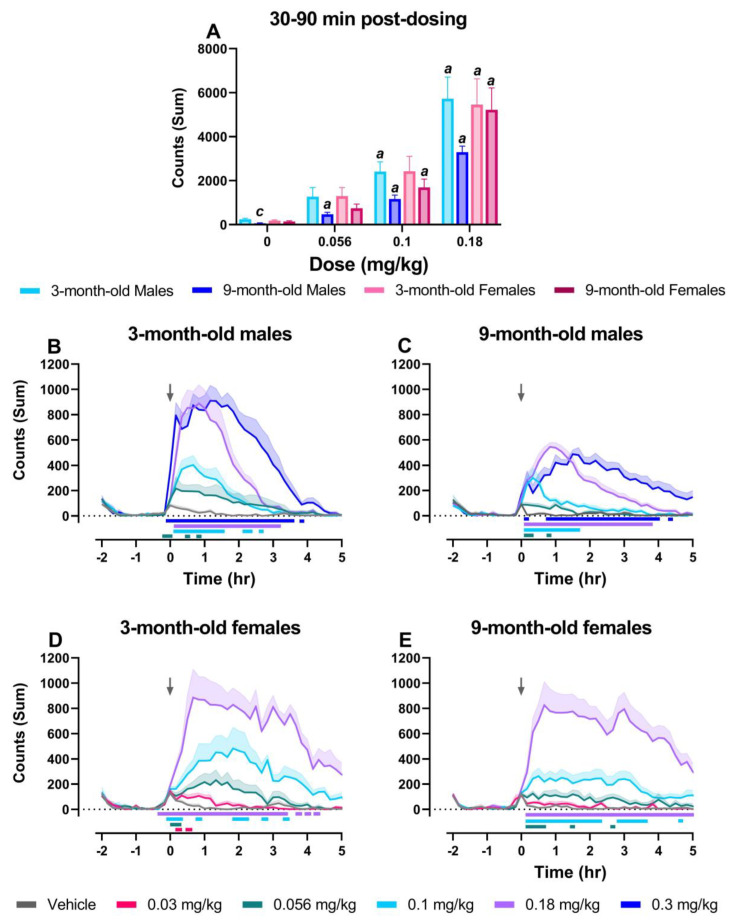
MK-801 dose-dependently increased locomotor activity in all groups. For direct group comparisons, each individual’s locomotor activity counts in the 30–90 min post-dosing period were summed and graphed as a group mean ± SEM (**A**). The effects of MK-801 on locomotor activity over time are displayed as group means ± SEM of the summed activity counts in 10 min bins across the 7 h recording period for 3-month-old male (*n* = 8–9) (**B**), 9-month-old male (*n* = 7–8) (**C**), 3-month-old female (*n* = 6) (**D**), and 9-month-old female rats (*n* = 9) (**E**). MK-801 was administered at time point 0, denoted by an arrow. On the x-axis, −2 corresponds to ZT 0, and 5 corresponds to ZT 7. In (**A**), *p* < 0.05; *a*, compared to group’s respective vehicle condition; *c*, compared to 3-month-old male rats. In (**B**–**E**), horizontal colored lines matching the respective dose color represent the 10 min bins at which MK-801-treated groups were significantly different from vehicle-treated groups (*p* < 0.05).

Lastly, we examined differences in locomotor activity because NMDA receptor antagonist-induced hyperlocomotion is commonly used as a correlate for positive symptoms of schizophrenia and used to predict antipsychotic response in preclinical models [8]. A three-way mixed ANOVA directly comparing summed locomotor activity in the 30–90 min post-dosing period revealed a main effect of dose (F_1.56, 42.59_ = 66.71; *p* < 0.0001) and age (F_1, 28_ = 6.241, *p* < 0.05), but no effect of sex and no significant interactions. Two-way ANOVAs were conducted to assess dose and age at each level of sex (males and females). In male rats, there was a main effect of dose (F_1.680, 32.48_ = 33.65, *p* < 0.0001) and age (F_1, 58_ = 12.39, *p* < 0.001) (Figure 4A). Post hoc tests revealed significant increases in locomotor activity relative to their respective vehicle condition in both 3-month-old (0.1 and 0.18 mg/kg) and 9-month-old (0.056–0.18 mg/kg) male rats. Locomotor activity was also significantly lower in 9-month-old male rats compared to 3-month-old male rats following vehicle administration, but this could in part be attributed to great differences in weight/size between 3- and 9-month-old males (see Table S1). In female rats, there was only a main effect of dose (F_1.402, 18.23_ = 33.50, *p* < 0.0001); here, MK-801-induced increases in locomotor activity relative to each group’s respective vehicle condition were found in both 3-month-old (0.18 mg/kg) and 9-month-old (0.1 and 0.18 mg/kg) female rats (Figure 4A).

Looking at dose-related changes in each group over time, there was a main effect of dose, time, and a dose-by-time interaction on locomotor activity, with all groups displaying dose-dependent increases in locomotor activity counts, assessed as a sum in 10 min bins across the full recording period (Figure 4B–E; for statistics, see Table 2). Post hoc analysis revealed significant increases at all tested doses in 3-month-old and 9-month-old male rats (0.056–0.3 mg/kg; Figure 4B,C) as well as in 3-month-old female rats (0.03–0.18 mg/kg; Figure 4D) at multiple 10 min bins following MK-801 administration; effects dissipated by hour 5. In 9-month-old female rats, post hoc results yielded similar results, with reported increases in locomotor activity at the 0.056, 0.1, and 0.18 mg/kg dose; significant increases following administration of 0.18 mg/kg MK-801 were still present 5 h post-dosing (Figure 4E).

## 3. Discussion

Using EEG to assess neuronal oscillatory activity represents a translational approach to investigate alterations in neurobiology or pathological progression in neuropsychiatric disorders. Aberrant elevations in resting-state gamma power have been identified in patients with schizophrenia and correspond to positive symptoms and cognitive impairment [22,23,24,25]. Importantly, gamma power is directly impacted by changes in NMDA receptor function, and NMDA receptor antagonists including MK-801 increase gamma power and disrupt cognition [30,34]. Although sex- and age-related differences are relevant factors influencing the pathophysiology of schizophrenia, how these variables affect NMDA receptor function has been largely overlooked in preclinical studies as well as in clinical trials [35]. In the present studies, consistent with prior literature, MK-801 produced dose-dependent changes in several frequency bands in 3-month-old male rats, with the strongest effects in the high gamma frequency band [27]. However, differences in the magnitude and overall temporal pattern of effects on gamma power were complex and further support that both age and sex influence NMDA receptor function. Notably, altered responsivity to MK-801 with regard to peak elevations, dose and time course may shed light on putative mechanistic alterations underlying the present age, sex, and age-by-sex differences.

These data suggest potential age-related changes that may be affected by altered NMDA receptor expression or activity on PV interneurons. According to the pyramidal–interneuron gamma (PING) mechanism, gamma oscillations are generated by the activity of cortical PV interneurons and excitatory pyramidal neurons [26,27,36,37]. Pyramidal cells provide excitatory glutamatergic input on PV interneurons, and excitatory currents are in turn synchronized as a result of feedback inhibition from these PV interneurons [28,36,38,39]. The pharmacological blockade of NMDA receptors predominantly reduces the activity of PV interneurons, leading to the disinhibition of pyramidal cells, increased pyramidal cell signaling, and thereby increased gamma power [27,37]. Alternate theories, however, suggest only interneurons are responsible for generating gamma oscillations (interneuron gamma models; ING) [36,39]. Importantly, increased basal gamma power and blunted elevations in response to MK-801 have been reported following genetic knockdown of NMDA receptors on PV cells in mice [28]. In the present studies, 9-month-old female rats displayed a similar profile to these mice, also showing higher basal gamma power compared to all other groups (Figure 1G) and a blunted maximum increase following MK-801 administration compared to 3-month-old female rats (Figure 3B). Given these similarities, this may suggest that 9-month-old female rats have reduced NMDA receptor expression on PV interneurons relative to other groups. NMDA receptors are heterotetrameric and are composed of two obligatory GluN1 subunits [40] and two GluN2 (splice variants including GluN2A-D) or GluN3 subunits [41]. Advanced aging has been associated with a decline in cortical GluN1 subunit expression [40,42,43], decreased [^3^H]MK-801 binding to the NMDA receptor complex in both rodent and human studies [44,45], and impaired NMDA receptor function on PV interneurons in male rats [42,46]. However, to our knowledge, similar functional studies have not been conducted in female rats. Interestingly, while 9-month-old male rats did not display an elevated baseline high gamma power, they showed a blunted maximal increase following MK-801 administration relative to 3-month-old males (Figure 3B). Taken together, our results suggest that by 9-months of age rats may be experiencing shifts in NMDA receptor expression leading to age-related functional differences. While speculative, this shift in expression may be more progressed in 9-month-old females, contributing to the marked differences in this group; hormonal factors influencing NMDA receptor subunit expression may underscore some of these age- and sex-related differences (see below).

Additionally, since gamma power reflects the balance of cortical excitatory (E) and inhibitory (I) activity, any alterations in E–I balance may contribute to changes in basal and evoked gamma oscillations. Following the administration of low to moderate doses of MK-801, increases in gamma power occur, likely driven by disinhibition of glutamatergic pyramidal neurons. However, MK-801 has previously been shown to have a biphasic effect on cortical gamma power [27,47]. As NMDA receptors are expressed on both PV interneurons and pyramidal neurons [26,27,37], administration of high doses of MK-801 results in a greater impact of an NMDA receptor blockade on pyramidal neurons, which dampens disinhibition-related hyperexcitability and prevents increases in gamma power [27,47]. Thus, by evaluating a broad dose range of MK-801, we examined sex- and age-related differences in E–I balance (Figure 3A). Importantly, given known sex differences in the metabolism of MK-801 [48,49], we focused on the initial 30–90 min period following administration. As shown in Figure 3C–F, all groups showed a rapid increase in gamma power at lower tested doses of MK-801 (0.03–0.1 mg/kg) following administration, with the exception of 9-month-old females, which did not show a large increase at any dose tested. At the higher tested doses (0.18 and 0.3 mg/kg), 3-month-old male rats showed a blunted response, followed by a delayed increase in gamma power around 90 min after administration. Nine-month-old male rats began to show this shift towards a delayed increase in gamma power at 0.3 mg/kg MK-801. Three-month-old females also began to show a blunted and delayed increase in gamma power following administration of 0.18 mg/kg MK-801. A more drastic blunted response was seen (data not shown) in a small subset of 3-month-old females (0.3 mg/kg) and 9-month-old males (0.56 mg/kg), but full examination was not completed due to visible adverse effects. Interestingly, 9-month-old female rats demonstrated a sustained blunted effect following administration of 0.18 mg/kg MK-801. Collectively, as shown in Figure 3A, MK-801 demonstrated a biphasic dose response. At the 0.18 mg/kg dose, 3-month-old male rats did not have a significant elevation in the 30–90 min period compared to the vehicle condition, but elevations were significant in 9-month-old male rats, suggesting possible age-related alterations in E–I balance in males. Increases in gamma power associated with high doses at a later time point were likely attributed to lower circulating brain concentrations of MK-801 as a product of drug metabolism and elimination.

Group-related differences were also observed in beta and alpha power (see Supplementary Materials), which are additional waveforms disrupted in schizophrenia [50,51,52,53,54]. Briefly, age-related differences were identified as 9-month-old females experienced larger decreases in beta power at moderate doses (0.1 mg/kg) compared to 3-month-old females. Further, peak increases in alpha power occurred at lower doses (0.1 mg/kg) in 3-month-old males compared to 9-month-old males (0.18 mg/kg). While the underlying mechanisms contributing to these effects are not fully understood, findings suggest there are additional waveforms that may be sensitive to individual differences in NMDA receptor function and further support sex- and age-dependent alterations in E–I balance.

Collectively, the present data suggest age-related differences in response to MK-801 are more prominent in females than males. Despite the well-established relationship between the menopause transition and schizophrenia in humans [1,2,55], limited preclinical studies have examined hormonal changes as underlying factors impacting NMDA receptor function. While the present studies did not examine the estrous phase or hormone concentrations, they are important considerations for future studies, and some results from the 9-month-old female rats may be explained by circulating levels of hormones including 17β-estradiol. Unlike the human menopause transition, which is characterized by follicular depletion accompanied by a shift in several hormone levels, including a reduction in overall circulating levels of 17β-estradiol and progesterone [5,56], middle-aged rats do not experience similar levels of follicular depletion and, thus, do not undergo menopause. Instead, female rats at ~9–12 months of age undergo a series of hormonal changes and experience irregular estrous cycles, termed estropause. In this timeframe, rats experience cyclic irregularities and commonly enter a persistent estrus phase associated with moderate to high levels of circulating 17β-estradiol [56,57]. While speculative, changes in 17β-estradiol levels in 9-month-old female rats may have impacted MK-801-induced changes in gamma power. Research supports a role for 17β-estradiol on NMDA receptor subunit expression. Cortical NMDA receptor expression fluctuates across the estrous cycle, and the luteal phase (metestrus/diestrus) as well as overall 17β-estradiol deprivation in female rats leads to decreased NMDA receptor subunit gene and protein expression [58,59,60,61,62]. GluN2A is the primary GluN2 subunit expressed in NMDA receptors in adulthood [63,64] and is the most abundant subunit on PV interneurons as demonstrated through genetic knockdown of GluN2A [65]. While not tested in the present study, GluN2A-preferring, but not GluN2B-selective, antagonists induce elevations in gamma power [29]. Interestingly, estrous cycle in females has been shown to impact the response to NMDAR antagonists in a GluN2A-dependent manner on PV interneurons [62]; thus, hormonal influence on synaptic GluN2A expression may underscore differences between 3- and 9-month-old females. Overall, based on present results, future studies that systematically evaluate the presence and absence of 17β-estradiol and other hormones on NMDA receptor function are needed.

Lastly, we assessed locomotor activity in all groups, which is a widely used preclinical readout for psychotomimetic-like effects and is frequently used to predict an antipsychotic response [8,66,67]. Though not significant, 9-month-old male rats demonstrated lower levels of home-cage locomotion at baseline and significantly lower levels of home-cage locomotion following vehicle administration, which is likely attributed to a significantly greater overall weight (see Table S1). Interestingly, MK-801 produced similar dose-dependent increases in locomotor activity in all groups. Previous research using an open-field assessment has found female rats to be more sensitive to the locomotor effects of MK-801 [68,69], though these results did not translate to the present studies, where rats were assessed in their familiar home environment. Importantly, our lack of group differences in locomotor activity despite significant group differences in gamma power suggests a disconnect between behavioral (hyperlocomotor) and functional screens. Differences identified between groups when using qEEG suggest locomotor activity may not be as sensitive to key individual variables. Gamma power is also not directly influenced by increases in locomotor activity, thus serving as a more direct measure of brain function [48]. Thus, qEEG represents a more translational approach in preclinical models to identifying biomarkers of brain function that are sensitive to individual age- and sex-related differences. 

### Conclusions

In summary, multiple factors can lead to a shift in E–I balance and thereby influence gamma power, including differences in NMDA receptor expression [42,43,62,70], glutamate neurotransmission [71,72], GABA signaling [73,74], and dendritic spine density [75,76]. The present evidence supports age- and sex-related differences in NMDA receptor function, primarily in female rodents. This suggests that these variables may influence underlying pathological factors in schizophrenia and support incorporating qEEG into preclinical and clinical studies as a translational measure that could be used to stratify patients and predict treatment efficacy across a heterogeneous population. Furthermore, the relevance of these studies may extend beyond schizophrenia. In particular, there are vast similarities between symptoms of schizophrenia and neuropsychiatric symptoms (NPS) associated with Alzheimer’s disease (AD). NPS in AD and related dementias affect around 60% of patients, with females demonstrating more severe symptoms, suggesting a plausible overlap in their etiology and risk factors [77,78,79,80]. While AD is a complex, multifactorial disease, evidence supports the hypothesis that NMDA receptor hypofunction may be one pathophysiological underpinning [46,77,81], and patients with AD have reduced gene expression of the GluN1 subunit compared to age-matched controls [40]. However, studies assessing the relationship between qEEG and NPS are still in their infancy and have not considered sex differences [82].

Ultimately, studies are needed to investigate additional factors underlying the present findings. For example, age- and sex-related differences in dopamine concentrations have been reported following NMDA receptor manipulation [83,84]. Furthermore, the present studies need to be extended to post-menopausal and geriatric animal models (i.e., 18–22-month-old). Understanding how sex- and age-dependent neurobiological changes impact PV interneuron function and thus E–I balance can be a valuable step toward advancing therapeutic interventions in neuropsychiatric disorders, including schizophrenia and AD.

## 4. Materials and Methods

### 4.1. Animals

The female (3-month-old, *n* = 6; 9-month-old, *n* = 9) and male (3-month-old, *n* = 8–9; 9-month-old, *n* = 7–8) Sprague–Dawley rats used in this study were obtained from Envigo (Indianapolis, IN, USA) and were individually housed in opaque cages (8 in × 10 in × 8 in) in temperature-controlled (range: 70–74 °F) colony rooms. All rats had ad libitum access to food and water and were maintained on a 12/12 h light/dark cycle. All procedures conducted were in accordance with the Wake Forest University School of Medicine Animal Care and Use Committee and complied with the National Institutes of Health Guide for the Care and Use of Laboratory Animals (Approval Code: D16-00248 (A3391-01)). One 3-month-old male and one 9-month-old male rat did not complete the full dose–response study due to deteriorating EEG signal quality.

### 4.2. Drugs

(+) MK-801 hydrogen maleate (Sigma-Aldrich, St. Louis, MO, USA; 0.03–0.18 mg/kg, subcutaneous (SC) in females, 0.056–0.3 mg/kg SC in males) was dissolved in sterile saline as an aqueous solution and was administered at a volume of 1 mL/kg. Briefly, doses reflect (+) MK-801 hydrogen maleate; the adjusted dose range of MK-801 base (excluding hydrogen maleate) are 0.02–0.12 mg/kg in females and 0.04–0.2 mg/kg in males. Females were tested with a lower dose range than males given known sex differences in NMDA receptor antagonist metabolism [48,49]. The dose order of MK-801 followed a counterbalanced design with a minimum of 3 days between each test day.

### 4.3. Surgery

Rats were anesthetized with isoflurane (3–5% induction, 1–3% maintenance) and implanted with a subcutaneous transmitter (HD-S02; Data Sciences International [DSI], Minneapolis, MN, USA) as previously described [34,85]. In brief, transmitters were implanted subcutaneously near the dorsal flank, and four wires were tunneled towards the skull. Two exposed wires for EEG were looped and placed in contact with the dura through holes (1.2 mm drill burr tip) drilled at +2 mm anterior to bregma and +2 mm from the midline (frontal cortex) and at −6 mm posterior to bregma and −2 mm from the midline (contralateral occipital cortex); wires were secured, and holes were covered with dental cement (Dentsply Sirona, Charlotte, NC, USA). Additionally, two wires were placed antiparallel through the nuchal muscle to measure muscle activity (electromyography; EMG). Rats were treated with Alloxate (Pivetal, Liberty, MO, USA; analgesic; 1 mg/kg, SC) and Baytril (Elanco US Inc., Shawnee, KS, USA; antibiotic; 5 mg/kg, SC) and recovered from surgery for a minimum of 7 days prior to the start of EEG recordings and MK-801 administration.

### 4.4. EEG Recordings

All recordings occurred within each rat’s home cage in their housing room, and each recording was 7 h total beginning at the onset of the light cycle (Zeitgeber time 0, ZT0). Using a within-subject design, MK-801 was administered 2 h following the start of the recording, allowing each test day to have a 2 h baseline period, and continued for 5 h following MK-801 administration. EEG, EMG, locomotor activity (activity counts—influenced by distance and speed of animal movement around the receiver), and temperature were transmitted via telemetry to a receiver located beneath each rat’s home cage and to a computer for offline analysis.

### 4.5. Sleep Staging and Analysis

Ten-second epochs were manually designated as different sleep/wake stages based on accepted oscillatory patterns (wake, rapid eye movement [REM], non-REM [NREM]) or artifact using Neuroscore 3.0 Software (DSI) as previously described [34,85]. Artifact was characterized by abnormally high amplitude or signal dropout.

### 4.6. qEEG Spectral Power Analysis

Following sleep staging, the power spectrum was computed in 1 Hz bins from 0.5 to 100 Hz using a Fast Fourier Transform with a Hamming window and overlap ratio of 0.5 within each 10 s epoch (Neuroscore). Using custom MATLAB scripts, the individual frequencies (0.5–100 Hz) or frequency bands (delta [0.5–4 Hz], theta [4–8 Hz], alpha [8–12 Hz], sigma [12–16 Hz], beta [16–24 Hz], low gamma [30–50 Hz], and high gamma [50–100 Hz]) were subsequently separated by sleep state (wake, NREM, or REM) to identify state-dependent changes in absolute or relative power [34,85]. The data for each frequency or frequency band were averaged into 10 min, 60 min, or 90 min bins. This study primarily focused on changes in relative power (e.g., power within one frequency band as a percentage of total spectral power), specifically during awake periods due to the wake-promoting effects of MK-801 [86]. Baseline comparisons in absolute and relative power for all frequency bands were examined by averaging the 90 min period directly prior to dosing on each rat’s vehicle day. The 90 min baseline period was selected to exclude the first 30 min to avoid the effects of initial experimenter-induced arousal when the transmitters were turned on.

Within-session changes in response to each MK-801 dose are expressed as a percent change from each individual rat’s averaged, same-day 90 min baseline period. Full spectrum graphs depict the averaged percent change from baseline during the 30–90 min post-dosing period. This time period was selected to correspond to peak brain tissue concentrations of MK-801 [87]. Time-course graphs assessing individual frequency bands depicted the percent change from baseline in 10 min bins across the 7 h recording period. Direct group comparisons for the individual frequency bands (i.e., high gamma power) were determined by averaging each individual’s percent change from baseline during the 30–90 min post-dosing period. Furthermore, to determine the peak effects of MK-801-induced elevations on high gamma power, the percent change from baseline was examined in sliding 30 min windows throughout the 5 h post-dosing period, and the highest value for each individual rat regardless of dose was identified and averaged within each group. Lastly, locomotor activity was simultaneously collected, and activity counts were summed in 10 min or 60 min bins to correspond to all time points described above.

### 4.7. Statistical Analysis

Between-group comparisons for baseline relative power, baseline absolute power, baseline locomotor activity, and peak high gamma power were analyzed using a two-way analysis of variance (ANOVA) followed by Šídák’s multiple comparisons test. A mixed-effects two-way ANOVA followed by Dunnett’s post hoc test was used to analyze dose–effect relationships in each group alone for the full power spectrum, each frequency band over time, and locomotor activity over time. A three-way mixed ANOVA was used to directly compare the effects of sex (2 levels: male and female), age (2 levels: 3- and 9-month-old), and MK-801 dose (4 levels: vehicle, 0.056 mg/kg, 0.1 mg/kg, and 0.18 mg/kg) on each frequency band and on locomotor activity during the 30–90 min post-dosing period. Two-way ANOVAs followed by Šídák’s multiple comparison tests were then conducted to assess effects of dose and age within each frequency band and on locomotor activity. Significance was always defined as *p* < 0.05.

## Figures and Tables

**Table 1:** Statistics describing the outcomes of MK-801 on relative power spectral distribution.

Full Spectrum Statistics							
Figure	Source of Variation	DF	F	*p*	*	*Post Hoc* Results	Significant Frequencies (Hz)
**2A** **3-Month-Old** **Males**	Dose	2.7, 21.60	7.675	0.0015	**	Vehicle vs. 0.056 mg/kg Vehicle vs. 0.1 mg/kg Vehicle vs. 0.18 mg/kg Vehicle vs. 0.3 mg/kg	13–21, 32–99 6, 9, 14–20, 44–57 7, 10, 13–27 4, 6, 7, 14–31
	Frequency	1.81, 14.47	28.00	<0.0001	****		
	Interaction	4.25, 32.93	6.011	0.0008	***		
**2B** **9-Month-Old** **Males**	Dose	2.51, 17.58	6.055	0.0069	**	Vehicle vs. 0.056 mg/kg Vehicle vs. 0.1 mg/kg Vehicle vs. 0.18 mg/kg Vehicle vs. 0.3 mg/kg	26, 41, 43, 57, 59, 61, 62, 64–74, 76, 77, 79, 80, 82–85, 87–99 11–26, 34–99 8, 11–26, 28, 44–48, 50–99 11–26, 93, 94, 96–99
	Frequency	1.92, 13.42	25.79	<0.0001	****		
	Interaction	4.34, 27.08	9.510	<0.0001	****		
**2C** **3-Month-Old** **Females**	Dose	1.36, 6.80	6.645	0.0317	*	Vehicle vs. 0.03 mg/kg Vehicle vs. 0.056 mg/kg Vehicle vs. 0.1 mg/kg Vehicle vs. 0.18 mg/kg	5, 6, 16, 38–68 3–6, 12–22, 36, 38–93 2–6, 15–20, 52–88, 91, 92 5–7, 15–29, 50–91, 94
	Frequency	2.09, 10.47	27.57	<0.0001	****		
	Interaction	2.56, 12.80	4.082	0.0351	*		
**2D** **9-Month-Old** **Females**	Dose	1.95, 15.60	6.207	0.0108	*	Vehicle vs. 0.03 mg/kg Vehicle vs. 0.056 mg/kg Vehicle vs. 0.1 mg/kg Vehicle vs. 0.18 mg/kg	5, 34, 39, 40, 42 11–21, 37, 39–45 12–21 15, 16, 21–41
	Frequency	1.61, 12.90	10.14	0.0033	**		
	Interaction	3.49, 27.94	3.301	0.0291	*		

* *p* < 0.05; ** *p* < 0.01; *** *p* < 0.001; **** *p* < 0.0001.

**Table 2:** Statistics describing effects of MK-801 on relative high gamma power and home-cage activity.

Time-Course Statistics							
Figure	Source of Variation	DF	F	*p*	*	*Post Hoc* Results	Significant Time Points (10 Min Bin)
**3C** **3-Month-Old** **Males** High gamma	Dose	3.14, 25.14	12.42	<0.0001	****	Vehicle vs. 0.056 mg/kg Vehicle vs. 0.1 mg/kg Vehicle vs. 0.18 mg/kg Vehicle vs. 0.3 mg/kg	−120, 0, 40, 60, 70, 90–140, 160 70–190 90–190, 220–240, 280 110–260, 280
	Time	2.74, 21.91	15.37	<0.0001	****		
	Interaction	4.94, 38.02	6.45	0.0002	***		
**3D** **9-Month-Old** **Males** High gamma	Dose	2.43, 16.97	11.01	0.0005	***	Vehicle vs. 0.056 mg/kg Vehicle vs. 0.1 mg/kg Vehicle vs. 0.18 mg/kg Vehicle vs. 0.3 mg/kg	10, 50, 90, 150, 170, 220, 230, 250 10–160, 180, 200 230, 250 10, 30–260 90, 100, 120–300
	Time	2.16, 15.15	7.048	0.006	**		
	Interaction	3.48, 21.26	4.563	0.0102	*		
**3E** **3-Month-Old** **Females** High gamma	Dose	1.80, 8.98	18.35	0.0008	***	Vehicle vs. 0.03 mg/kg Vehicle vs. 0.056 mg/kg Vehicle vs. 0.1 mg/kg Vehicle vs. 0.18 mg/kg	50, 80, 290 30–220 −40, 10–200, 220–300 −40, 0, 50–300
	Time	2.11, 10.55	24.91	<0.0001	****		
	Interaction	4.10, 20.11	7.625	0.0006	***		
**3F** **9-Month-Old** **Females** High gamma	Dose	1.73, 13.86	6.45	0.0127	*	Vehicle vs. 0.03 mg/kg Vehicle vs. 0.056 mg/kg Vehicle vs. 0.1 mg/kg	100, 140, 220 150, 160, 240, 250 160, 200, 240, 280
	Time	1.67, 13.36	4.26	0.0428	*		
	Interaction	4.05, 27.95	3.94	0.0115	*		
**4B** **3-Month-Old** **Males** Activity	Dose	2.27, 18.14	27.04	<0.0001	****	Vehicle vs. 0.056 mg/kg Vehicle vs. 0.1 mg/kg Vehicle vs. 0.18 mg/kg Vehicle vs. 0.3 mg/kg	−10, 0, 30, 50 10–90, 130, 140, 160 10–190 0–210, 230
	Time	2.03, 16.23	64.56	<0.0001	****		
	Interaction	2.81, 21.61	11.72	0.0001	***		
**4C** **9-Month-Old** **Males** Activity	Dose	1.67, 11.68	43.18	<0.0001	****	Vehicle vs. 0.056 mg/kg Vehicle vs. 0.1 mg/kg Vehicle vs. 0.18 mg/kg Vehicle vs. 0.3 mg/kg	10, 20, 50 10–100 10–220 10, 40–240, 260
	Time	3.52, 24.62	39.48	<0.0001	****		
	Interaction	4.69, 28.59	15.18	<0.0001	****		
**4D** **3-Month-Old** **Females** Activity	Dose	1.68, 8.40	32.5	0.0002	***	Vehicle vs. 0.03 mg/kg Vehicle vs. 0.056 mg/kg Vehicle vs. 0.1 mg/kg Vehicle vs. 0.18 mg/kg	60 30, 40 20–40. 80, 150–180, 220, 260 0–270, 290
	Time	1.74, 8.68	21.02	0.0006	***		
	Interaction	288, 1380	10.08	<0.0001	****		
**4E** **9-Month-Old** **Females** Activity	Dose	1.44, 11.48	37.89	<0.0001	****	Vehicle vs. 0.056 mg/kg Vehicle vs. 0.1 mg/kg Vehicle vs. 0.18 mg/kg	10–40, 90, 160 10–140, 170–220, 280 10–300
	Time	2.58, 20.63	18.74	<0.0001	****		
	Interaction	3.36, 28.79	10.85	<0.0001	****		

* *p* < 0.05; ** *p* < 0.01; *** *p* < 0.001; **** *p* < 0.0001.

## Data Availability

The data presented in this study are available on request from the corresponding author.

## Supplementary Materials

The following supporting information can be downloaded at: https://www.mdpi.com/article/10.3390/ph17020237/s1, Figure S1. There were no group differences in absolute power during the baseline period; Figure S2. MK-801 significantly affects all spectral power bands in 3-month-old male rats; Figure S3. MK-801 differentially affects spectral power bands in 9-month-old male rats; Figure S4. MK-801 differentially affects spectral power bands in 3-month-old female rats; Figure S5. MK-801 affects spectral power bands in the 12–50 (sigma, beta, low gamma) Hz range in 9-month-old female rats; Figure S6. MK-801-induced differences in relative power; Table S1. Average weights of rats in each group on first dose day; Table S2. Statistics for supplementary Figures S1 and S2; Table S3. Statistics for supplementary Figures S3 and S4.
