# Supplementary material for: Use of Quantitative Electroencephalography to Inform Age- and Sex-Related Differences in NMDA Receptor Function Following MK-801 Administration
Source: Pharmaceuticals (Basel). 2024 Feb 11;17(2):237. doi: 10.3390/ph17020237 (PMC10892193; doi:10.3390/ph17020237)
Supplement: Supplementary file 1 [file pharmaceuticals-17-00237-s001.zip › pharmaceuticals-2857693-supplementary.pdf]

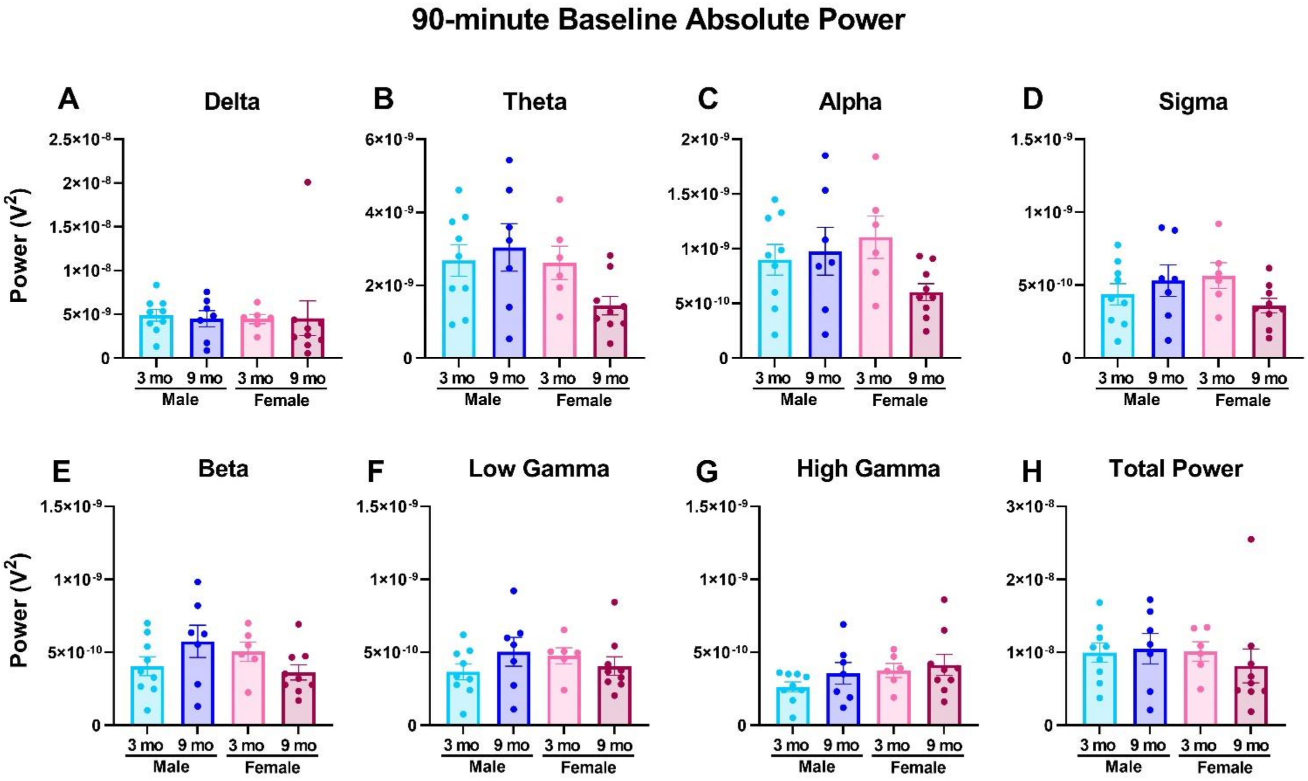

Figure S1. There were no group differences in absolute power during the baseline period. qEEG data are shown as a group mean ±SEM of absolute (A-G) or total (H) power during the 90 minute baseline period on each individuals' respective vehicle day. Data are summed across the delta [0.5–4 Hz] (A), theta [4–8 Hz] (B), alpha [8–12 Hz] (C), sigma [12–16 Hz] (D), beta [16–24 Hz] (E), low gamma [30–50 Hz] (F), high gamma [50–100 Hz] (G) frequency bands and summed total power [0.5-100 Hz] (H) to compare 3-month-old male (n=9) and female (n=6) and 9-month-old male (n=7) and female (n=9) rats; circles represent individual datapoints.

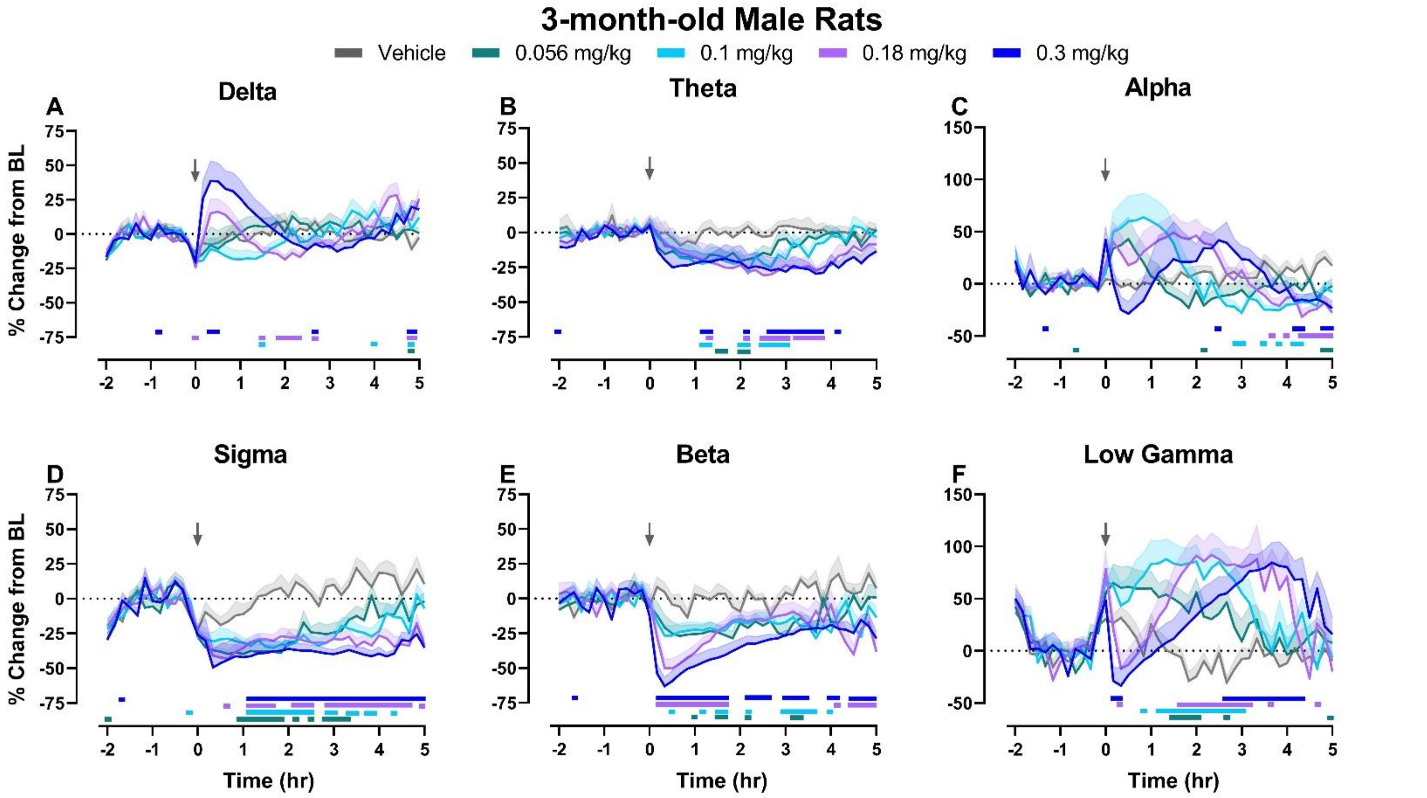

Figure S2. MK-801 significantly affects all spectral power bands in 3-month-old male rats. Time course effects of MK-801 are displayed as group means ±SEM (n=8-9) as the percent change from 90-min baseline in 10-minute bins across the 7-hour recording period on delta (A) theta (B) alpha (C) sigma (D), beta (E), and low gamma (F) in 3-month-old male rats. MK-801 was administered at time point 0, represented by an arrow. On the x-axis, -2 corresponds to ZT 0 and 5 corresponds to ZT 7. Horizontal colored lines matching the respective dose color represent the 10-minute bins at which MK-801-treated groups were significantly different from vehicle-treated groups.

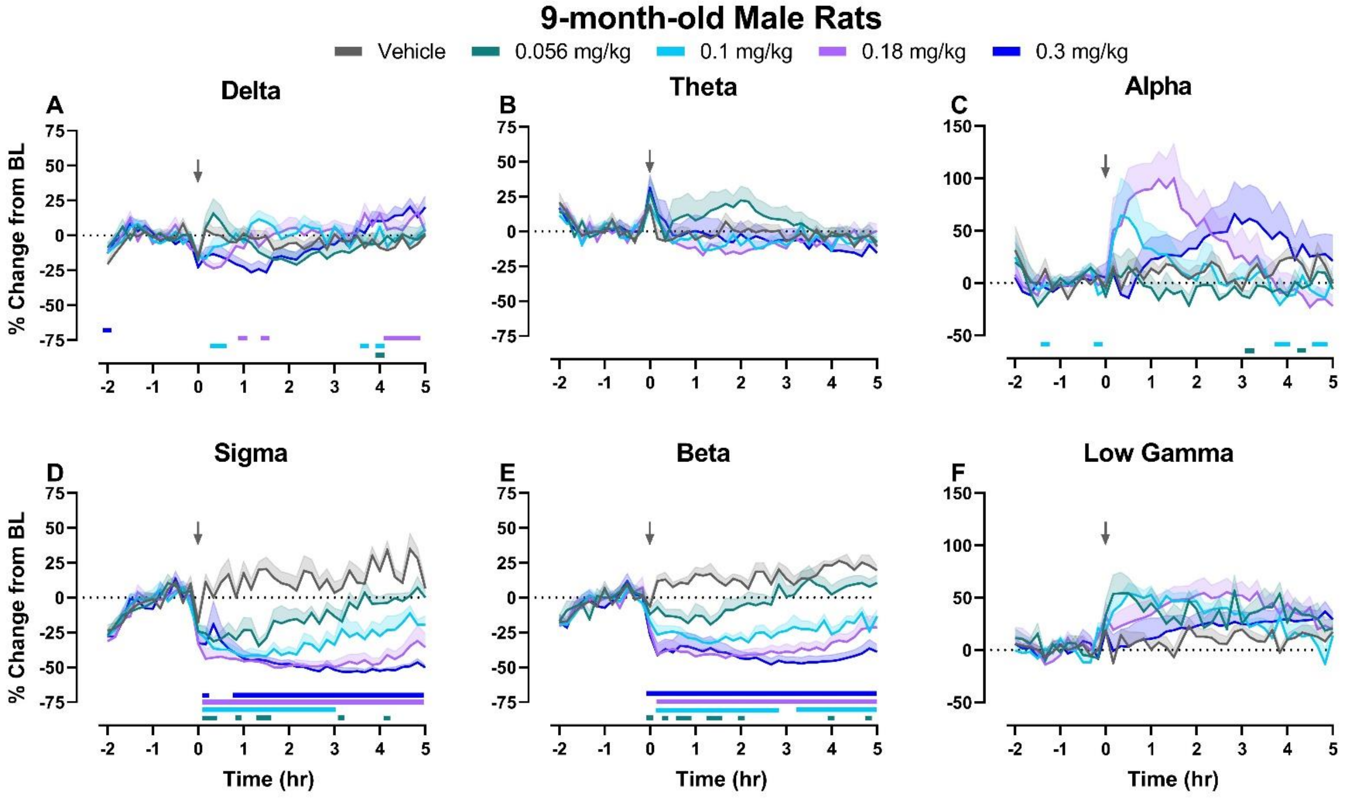

Figure S3. MK-801 differentially affects spectral power bands in 9-month-old male rats. Time course effects of MK-801 are displayed as group means ±SEM (n=7-8) as the percent change from 90-min baseline in 10-minute bins across the 7-hour recording period on delta (A) theta (B) alpha (C) sigma (D), beta (E), and low gamma (F) in 9-month-old male rats. MK-801 was administered at time point 0, represented by an arrow. On the x-axis, -2 corresponds to ZT 0 and 5 corresponds to ZT 7. Horizontal colored lines matching the respective dose color represent the 10-minute bins at which MK-801-treated groups were significantly different from vehicle-treated groups.

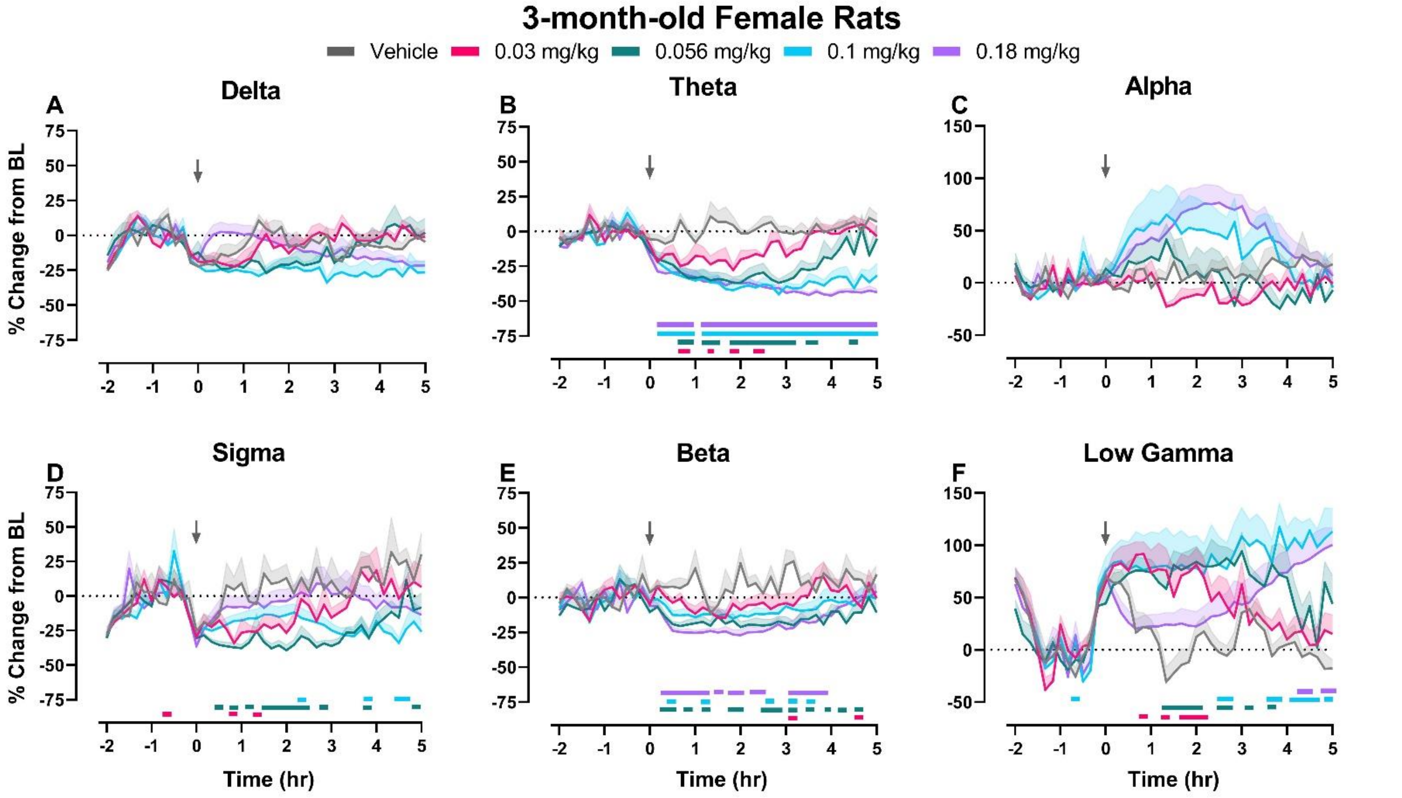

Figure S4. MK-801 differentially affects spectral power bands in 3-month-old female rats. Time course effects of MK-801 are displayed as group means ±SEM (n=6) as the percent change from 90-min baseline in 10-minute bins across the 7-hour recording period on delta (A) theta (B) alpha (C) sigma (D), beta (E), and low gamma (F) in 3-month-old female rats. MK-801 was administered at time point 0, represented by an arrow. On the x-axis, -2 corresponds to ZT 0 and 5 corresponds to ZT 7. Horizontal colored lines matching the respective dose color represent the 10-minute bins at which MK-801-treated groups were significantly different from vehicle-treated groups.

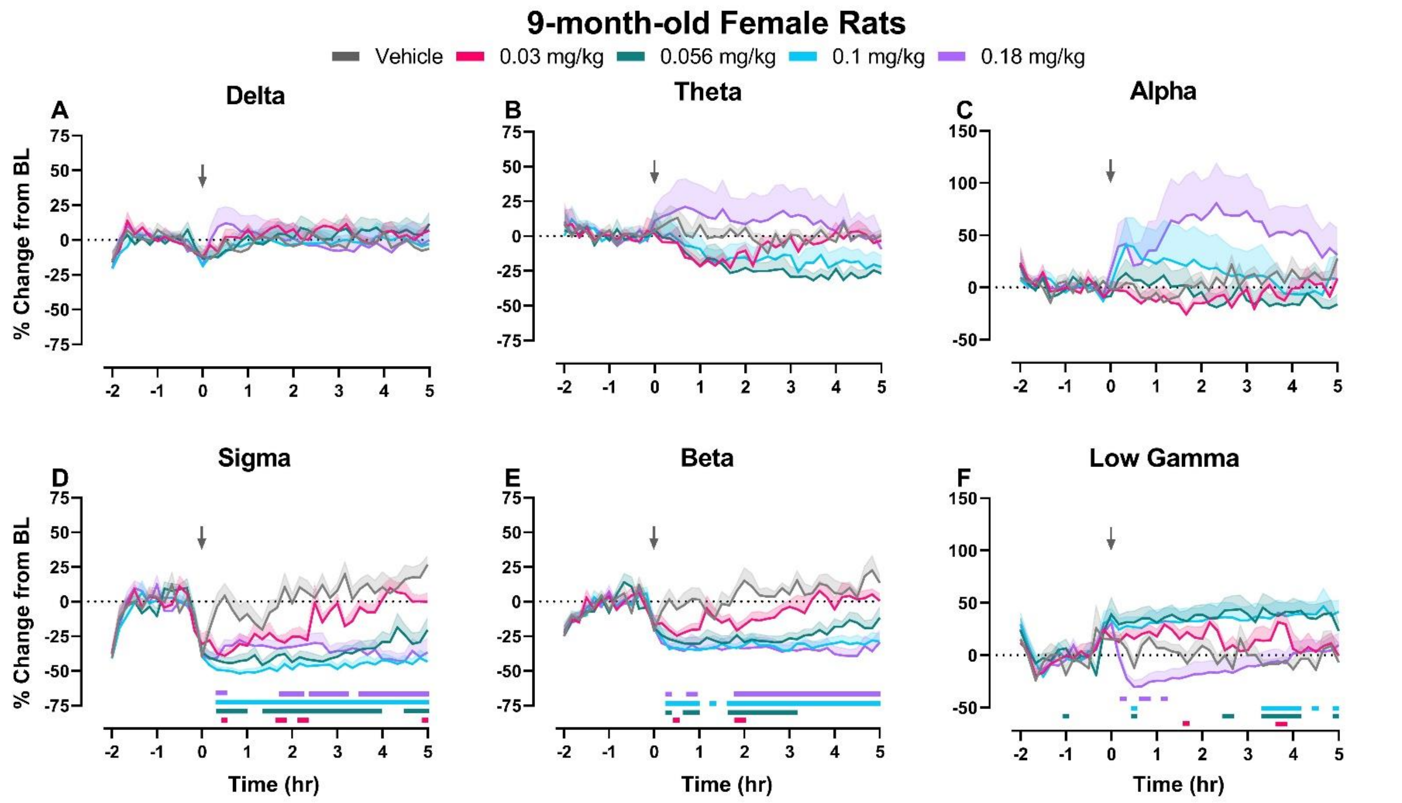

Figure S5. MK-801 affects spectral power bands in the 12-50 (sigma, beta, low gamma) Hz range in 9-month-old female rats. Time course effects of MK-801 are displayed as group means ±SEM (n=9) as the percent change from 90-min baseline in 10-minute bins across the 7-hour recording period on delta (A) theta (B) alpha (C) sigma (D), beta (E), and low gamma (F) in 9-month-old female rats. MK-801 was administered at time point 0, represented by an arrow. On the x-axis, -2 corresponds to ZT 0 and 5 corresponds to ZT 7. Horizontal colored lines matching the respective dose color represent the 10-minute bins at which MK-801-treated groups were significantly different from vehicle-treated groups.

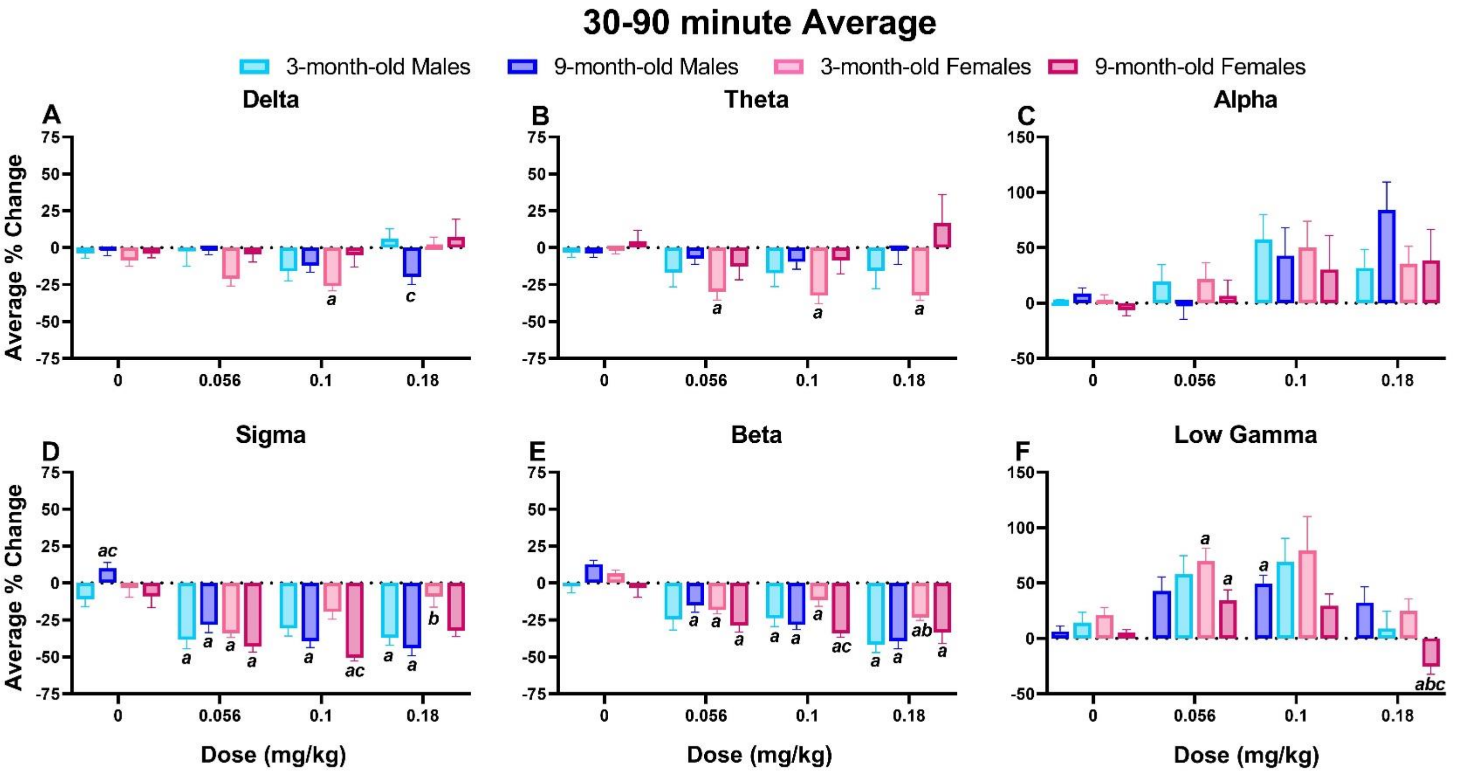

Figure S6. MK-801-induced differences in relative power. For direct group comparisons in response to MK-801, each individual's percent change from baseline in the 30-90 min post-dosing period was averaged and graphed as a group mean ±SEM to assess the effects of MK-801 administration on delta **(A)**, theta **(B)**, alpha **(C)**, sigma **(D)**, beta **(E)**, and low gamma power **(F)**. A three-way ANOVA was run for each graph. For delta, there was a main effect of dose ($F_{2.522, 68.95}$= 3.962, p<0.05) and an dose x age interaction ($F_{3, 82}$= 2.89, p<0.05) **(A)**. For theta, there was main effect of dose ($F_{1.979, 54.10}$= 5.516, p<0.05) and age ($F_{1, 28}$= 4.501, p<0.05) and a dose x age interaction ($F_{3, 82}$= 3.391, p<0.05) **(B)**. For alpha, there was a main effect of dose ($F_{2.351, 64.27}$= 9.345, p= 0.0001) **(C)**. For sigma, there was a main effect of dose ($F_{2.576, 70.41}$= 40.89, p<0.0001) and age ($F_{1, 28}$= 4.599, p<0.05) and a significant dose x sex ($F_{3, 82}$= 6.284, p<0.001), dose x age ($F_{3, 82}$= 7.179, p<0.001), and age x sex ($F_{1, 28}$= 11.12, p<0.005) interaction **(D)**. For beta, there was a main effect of dose ($F_{2.716, 74.23}$= 46.82, p<0.0001) and a significant age x sex interaction ($F_{1, 28}$= 8.608, p<0.05) **(E)**. Lastly, for low gamma there was a main effect of dose ($F_{2.761, 75.47}$= 19.55, p<0.0001) and age ($F_{1, 28}$= 5.256, p<0.05) but no interactions **(F)**. p<0.05, *a*, compared to group's respective vehicle condition, *b,* compared to male rats of the same age and *c*, compared to 3-month-old rats of the same sex.

Supplementary Table S1. Average weights of rats in each group on first dose day

	3-month-old Males	9-month-old Males	3-month-old Females	9-month-old Females
**Average Weight**	325.8	776.9	224.8	288.9
**Std. Deviation**	9.4	168.3	8.7	10.9

Supplementary Table S2. Statistics for supplementary figures S1 and S2

Timecourse Statistics (Males)							
**Figure**	**Source of Variation**	**DF**	**F**	**p**	*****	***Post hoc* results**	**Significant Timepoints (10-minute bin)**
**3-month-old Males**							
**S1A** Delta	Dose	3.13, 25.01	2.383	0.0912	ns	Vehicle vs 0.056 mg/kg	290
	Time	3.31, 26.45	4.628	0.0083	**	Vehicle vs 0.1 mg/kg	90, 240, 290
	Interaction	4.67, 36.04	3.794	0.0083	**	Vehicle vs 0.18 mg/kg<br>Vehicle vs 0.3 mg/kg	0, 90, 110-140, 160, 290, 300<br>-50, 20, 30, 160, 290, 300
**S1B** Theta	Dose	1.98, 15.80	10.48	0.0013	**	Vehicle vs 0.056 mg/kg	90, 100, 120, 130
	Time	1.71, 13.70	4.033	0.0471	*	Vehicle vs 0.1 mg/kg	70, 80, 120, 130, 150-180
	Interaction	4.62, 35.42	1.631	0.1813	ns	Vehicle vs 0.18 mg/kg<br>Vehicle vs 0.3 mg/kg	80, 130, 150-170, 190-230<br>-120, 70, 80, 130, 160-230, 250
**S1C** Alpha	Dose	2.84, 22.68	0.668	0.572	ns	Vehicle vs 0.056 mg/kg	-40, 130, 290, 300
	Time	2.14, 17.08	3.603	0.047	*	Vehicle vs 0.1 mg/kg	170, 180, 210-230, 250, 260
	Interaction	3.84, 29.54	4.296	0.008	**	Vehicle vs 0.18 mg/kg<br>Vehicle vs 0.3 mg/kg	220, 240, 260, 270, 280-300<br>-80, 150, 250, 260, 290, 300
**S1D** Sigma	Dose	3.06, 24.48	18.73	<0.0001	****	Vehicle vs 0.056 mg/kg	60-110, 130, 150, 170-200
	Time	3.74, 29.91	16.88	<0.0001	****	Vehicle vs 0.1 mg/kg	-10, 70-150, 170, 180, 200, 210, 230, 240, 260
	Interaction	5.27, 40.49	3.10	0.017	*	Vehicle vs 0.18 mg/kg<br>Vehicle vs 0.3 mg/kg	40, 70-100, 130-150, 170-280, 300<br>-100, 70-300
**S1E** Beta	Dose	2.85, 22.82	16.11	<0.0001	****	Vehicle vs 0.056 mg/kg	60, 70, 90, 130, 190, 200
	Time	3.86, 30.84	11.86	<0.0001	****	Vehicle vs 0.1 mg/kg	30, 70, 90, 100, 130, 180-220, 240
	Interaction	4.76, 36.63	2.90	0.0282	*	Vehicle vs 0.18 mg/kg<br>Vehicle vs 0.3 mg/kg	10-100, 250, 270-300<br>-100, 10-100, 130-160, 180-210, 240, 250, 270-300
**S1F** Low Gamma	Dose	2.63, 21.03	7.941	0.0014	**	Vehicle vs 0.056 mg/kg	90-120, 160, 300
	Time	3.83, 30.67	7.851	0.0002	***	Vehicle vs 0.1 mg/kg	50, 70-180
	Interaction	4.98, 38.33	4.705	0.0019	**	Vehicle vs 0.18 mg/kg<br>Vehicle vs 0.3 mg/kg	20, 100-190, 220, 280<br>10, 20, 160-260
**9-month-old Males**							
**S2A** Delta	Dose	1.96, 13.68	1.030	0.3813	ns	Vehicle vs 0.056 mg/kg	240
	Time	3.76, 26.29	3.819	0.0156	*	Vehicle vs 0.1 mg/kg	10, 20, 220, 240
	Interaction	5.38, 32.79	3.223	0.0159	*	Vehicle vs 0.18 mg/kg<br>Vehicle vs 0.3 mg/kg	60, 90, 250-290<br>-120
**S2B** Theta	Dose	2.15, 15.01	2.218	0.1409	ns		
	Time	2.83, 19.83	3.910	0.0257	*		
	Interaction	4.63, 28.25	1.537	0.2133	ns		
**S2C** Alpha	Dose	1.56, 10.91	2.131	0.17	ns	Vehicle vs 0.056 mg/kg	190, 260
	Time	2.11, 14.81	3.279	0.0641	ns	Vehicle vs 0.1 mg/kg	-80, -10, 230, 240, 280, 290
	Interaction	3.30, 20.14	3.352	0.0359	*		
**S2D** Sigma	Dose	2.48, 17.34	45.27	<0.0001	****	Vehicle vs 0.056 mg/kg	10, 20, 50, 80, 90, 190, 250
	Time	4.16, 29.12	18.83	<0.0001	****	Vehicle vs 0.1 mg/kg	10-180, 200, 220-290
	Interaction	4.55, 27.73	6.013	0.0009	***	Vehicle vs 0.18 mg/kg<br>Vehicle vs 0.3 mg/kg	10-300<br>10, 50-300
**S2E** Beta	Dose	2.51, 17.54	44.04	<0.0001	****	Vehicle vs 0.056 mg/kg	0, 20, 40, 50, 80, 90, 120, 240, 290
	Time	4.10, 28.68	13.15	<0.0001	****	Vehicle vs 0.1 mg/kg	10-170, 200-300
	Interaction	5.09, 31.03	7.211	0.0001	***	Vehicle vs 0.18 mg/kg<br>Vehicle vs 0.3 mg/kg	10-300<br>0-300
**S2F** Low Gamma	Dose	2.65, 18.52	2.495	0.0975	ns		
	Time	3.12, 21.82	9.171	0.0004	***		
	Interaction	4.19, 25.56	2.293	0.0845	ns		

*p<0.05; ** p<0.01; *** p<0.001; ****p<0.0001

Supplementary Table S3. Statistics for supplementary figures S3 and S4

Timecourse Statistics (Females)							
**Figure**	**Source of Variation**	**DF**	**F**	**p**	*****	***Post hoc* results**	**Significant Timepoints (10-minute bin)**
**3-month-old Females**							
**S3A** Delta	Dose	1.74, 8.70	6.239	0.0729	ns		
	Time	4.04, 20.17	3.699	0.0019	**		
	Interaction	3.97, 19.52	2.737	0.0587	ns		
**S3B** Theta	Dose	2.42, 12.08	44.16	<0.0001	****	Vehicle vs 0.03 mg/kg	40, 50, 80, 110, 140, 150
	Time	2.85, 14.24	16.01	<0.0001	****	Vehicle vs 0.056 mg/kg	40, 50, 70-90, 110-190, 210, 220, 270
						Vehicle vs 0.1 mg/kg	10-50, 70-300
	Interaction	3.41, 16.73	5.804	0.0053	**	Vehicle vs 0.18 mg/kg	10-50, 70-300
**S3C** Alpha	Dose	2.19, 10.94	3.306	0.0722	ns		
	Time	2.23, 11.16	5.002	0.0256	*		
	Interaction	3.62, 17.77	2.972	0.052	ns		
**S3D** Sigma	Dose	1.75, 8.77	5.218	0.0351	*	Vehicle vs 0.03 mg/kg	-40, 50, 80
	Time	2.75, 13.74	5.864	0.0096	**	Vehicle vs 0.056 mg/kg	30, 50-70, 90-150, 170, 230, 300
						Vehicle vs 0.1 mg/kg	10-50, 70-300
	Interaction	3.93, 19.27	2.586	0.0704	ns	Vehicle vs 0.18 mg/kg	10-50, 70-300
**S3E** Beta	Dose	2.63, 13.12	12.26	0.0006	***	Vehicle vs 0.03 mg/kg	190, 280
	Time	2.60, 13.01	4.067	0.0344	*	Vehicle vs 0.056 mg/kg	20, 30, 50, 70, 110,120,150-170,190, 210, 230, 250, 280
						Vehicle vs 0.1 mg/kg	30, 70, 160, 190, 210
	Interaction	3.48, 17.09	1.984	0.1484	ns	Vehicle vs 0.18 mg/kg	20-70, 90, 110, 120, 140, 150, 190-230
**S3F** Low Gamma	Dose	1.12, 5.59	5.375	0.0607	ns	Vehicle vs 0.03 mg/kg	50, 80, 100-130
	Time	2.77, 13.87	12.86	0.0003	***	Vehicle vs 0.056 mg/kg	80-120, 150, 160, 190, 220
						Vehicle vs 0.1 mg/kg	-40, 150, 160, 220, 230, 250-280, 300
	Interaction	3.87, 18.99	3.984	0.0171	*	Vehicle vs 0.18 mg/kg	260, 270, 290, 300
**9-month-old Females**							
**S4A** Delta	Dose	2.33, 18.67	0.258	0.8072	ns		
	Time	2.46, 19.70	2.094	0.142	ns		
	Interaction	5.04, 34.76	1.188	0.3351	ns		
**S4B** Theta	Dose	1.48, 11.84	2.613	0.1241	ns		
	Time	1.37, 10.94	1.032	0.3586	ns		
	Interaction	2.05, 14.16	2.069	0.1621	ns		
**S4C** Alpha	Dose	2.43, 19.42	2.420	0.1069	ns		
	Time	2.07, 16.57	1.615	0.2286	ns		
	Interaction	3.95, 27.22	2.053	0.1156	ns		
**S4D** Sigma	Dose	1.74, 13.90	18.56	0.0002	***	Vehicle vs 0.03 mg/kg	30, 100, 110, 130, 140, 300
	Time	3.72, 29.78	18.35	<0.0001	****	Vehicle vs 0.056 mg/kg	20-60, 80-240, 280-300
						Vehicle vs 0.1 mg/kg	20-300
	Interaction	4.82, 33.23	3.767	0.0088	**	Vehicle vs 0.18 mg/kg	20, 30, 110-130, 150-190, 210-300
**S4E** Beta	Dose	2.35, 18.77	15.69	<0.0001	****	Vehicle vs 0.03 mg/kg	30, 110, 120
	Time	3.42, 27.33	10.68	<0.0001	****	Vehicle vs 0.056 mg/kg	20, 40-60, 100-190, 210-230, 260, 280-300
						Vehicle vs 0.1 mg/kg	20-60, 80, 100-300
	Interaction	4.46, 30.77	3.335	0.019	*	Vehicle vs 0.18 mg/kg	20, 50, 60, 110-300
**S4F** Low Gamma	Dose	2.28, 18.22	13.05	0.0002	***	Vehicle vs 0.03 mg/kg	100, 220, 230
	Time	2.97, 23.73	3.201	0.042	*	Vehicle vs 0.056 mg/kg	-60, 30, 150, 160, 200-250, 300
						Vehicle vs 0.1 mg/kg	30, 200-250, 270, 300
	Interaction	5.19, 35.81	3.347	0.013	*	Vehicle vs 0.18 mg/kg	20, 40, 50, 70
*p<0.05; ** p<0.01; *** p<0.001; ****p<0.0001							
